# Peroxisome Proliferator-Activated Receptors-Alpha and Gamma Are Targets to Treat Offspring from Maternal Diet-Induced Obesity in Mice

**DOI:** 10.1371/journal.pone.0064258

**Published:** 2013-05-20

**Authors:** D'Angelo Carlo Magliano, Thereza Cristina Lonzetti Bargut, Simone Nunes de Carvalho, Marcia Barbosa Aguila, Carlos Alberto Mandarim-de-Lacerda, Vanessa Souza-Mello

**Affiliations:** Laboratory of Morphometry, Metabolism and Cardiovascular Disease, Biomedical Centre, Institute of Biology, State University of Rio de Janeiro, Rio de Janeiro, Brazil; The Chinese University of Hong Kong, Hong Kong

## Abstract

**Aim:**

The aim of the present study was to evaluate whether activation of peroxisome proliferator-activated receptor (PPAR)*alpha* and PPAR*gamma* by Bezafibrate (BZ) could attenuate hepatic and white adipose tissue (WAT) abnormalities in male offspring from diet-induced obese dams.

**Materials and Methods:**

C57BL/6 female mice were fed a standard chow (SC; 10% lipids) diet or a high-fat (HF; 49% lipids) diet for 8 weeks before mating and during gestation and lactation periods. Male offspring received SC diet at weaning and were subdivided into four groups: SC, SC/BZ, HF and HF/BZ. Treatment with BZ (100 mg/Kg diet) started at 12 weeks of age and was maintained for three weeks.

**Results:**

The HF diet resulted in an overweight phenotype and an increase in oral glucose intolerance and fasting glucose of dams. The HF offspring showed increased body mass, higher levels of plasmatic and hepatic triglycerides, higher levels of pro-inflammatory and lower levels of anti-inflammatory adipokines, impairment of glucose metabolism, abnormal fat pad mass distribution, higher number of larger adipocytes, hepatic steatosis, higher expression of lipogenic proteins concomitant to decreased expression of PPAR*alpha* and carnitine palmitoyltransferase I (CPT-1) in liver, and diminished expression of PPAR*gamma* and adiponectin in WAT. Treatment with BZ ameliorated the hepatic and WAT abnormalities generated by diet-induced maternal obesity, with improvements observed in the structural, biochemical and molecular characteristics of the animals' livers and epididymal fat.

**Conclusion:**

Diet-induced maternal obesity lead to alterations in metabolism, hepatic lipotoxicity and adverse liver and WAT remodeling in the offspring. Targeting PPAR with Bezafibrate has beneficial effects reducing the alterations, mainly through reduction of WAT inflammatory state through PPAR*gamma* activation and enhanced hepatic *beta*-oxidation due to increased PPAR*alpha*/PPAR*gamma* ratio in liver.

## Introduction

Obesity and comorbidities (metabolic syndrome, MS, leading to type 2 diabetes mellitus, DM2, cardiovascular disease, CVD, and non-alcoholic fatty liver disease, NAFLD) is due not only to environmental factors but also to maternal nutrition [1]. According to the Developmental Overnutrition Hypothesis, maternal overnutrition leads to permanent alterations in appetite control, neuroendocrine behavior and/or energetic metabolism in offspring during development, leading to obesity in adulthood even in the absence of excessive energy intake [2], [3].

WAT is able to express and secrete several cytokines called adipokines, such as leptin, adiponectin and resistin, and has a role in the secretion of the proinflammatory cytokines tumor necrosis factor (TNF)-*alpha*, interleukin (IL)-6, monocyte chemoattractant protein (MCP)-1 and plasminogen activator inhibitor (PAI)-1 [4]. Obesity and its outcomes are closely linked to an increase in both visceral WAT and adipocyte size, highly affecting the function of this organ [5]. Visceral fat accumulation is a cornerstone for the development of obesity-related pathologies, such as NAFLD, in contrast with subcutaneous WAT [6], [7].

NAFLD is the hepatic component of MS and is associated with obesity, insulin resistance (IR) and DM2 [8], [9]. As the condition progresses, NAFLD evolves to non alcoholic steatohepatitis (NASH) because of continuous inflammation and the peroxidation of lipids. NASH can progress to cirrhosis through hepatic fibrosis and advance to hepatocarcinoma [10], [11]. In addition, maternal diet during gestation and lactation can induce IR and NAFLD in offspring [3], [12].

Peroxisome proliferator-activated receptors (PPARs) are a family of transcription factors (TFs) existing in three isoforms: PPAR*alpha*, PPAR*beta* and PPAR*gamma*. They are closely linked to carbohydrate, protein, and lipid metabolism as well as to cellular proliferation [13], [14]. BZ is often used in clinical practice as a hypolipidemic drug. It was reported as a pan-PPAR agonist activating all three isoforms, but it preferentially activates PPAR*alpha* and PPAR*beta* [15], [16]. BZ has been demonstrated to prevent DM2 both in patients with CVD and in patients having undergone heart attack [17]. Furthermore, it has been shown to result in improvement of NASH in an animal model [18]. Therefore, the present study was undertaken to evaluate whether Bezafibrate could attenuate alterations in the livers and WAT of male mice offspring from diet-induced obese dams.

## Materials and Methods

### Animals and diet

This study was carried out in strict accordance with the recommendations in the Guide for the Care and Use of Laboratory Animals of the National Institutes of Health (NIH Publication number 85-23, revised 1996). The protocol was approved by Ethics Committee for Animal Experimentation of the State University of Rio de Janeiro (Protocol Number CEUA/012/2011). All efforts were made to minimize suffering.

**Figure 1:**
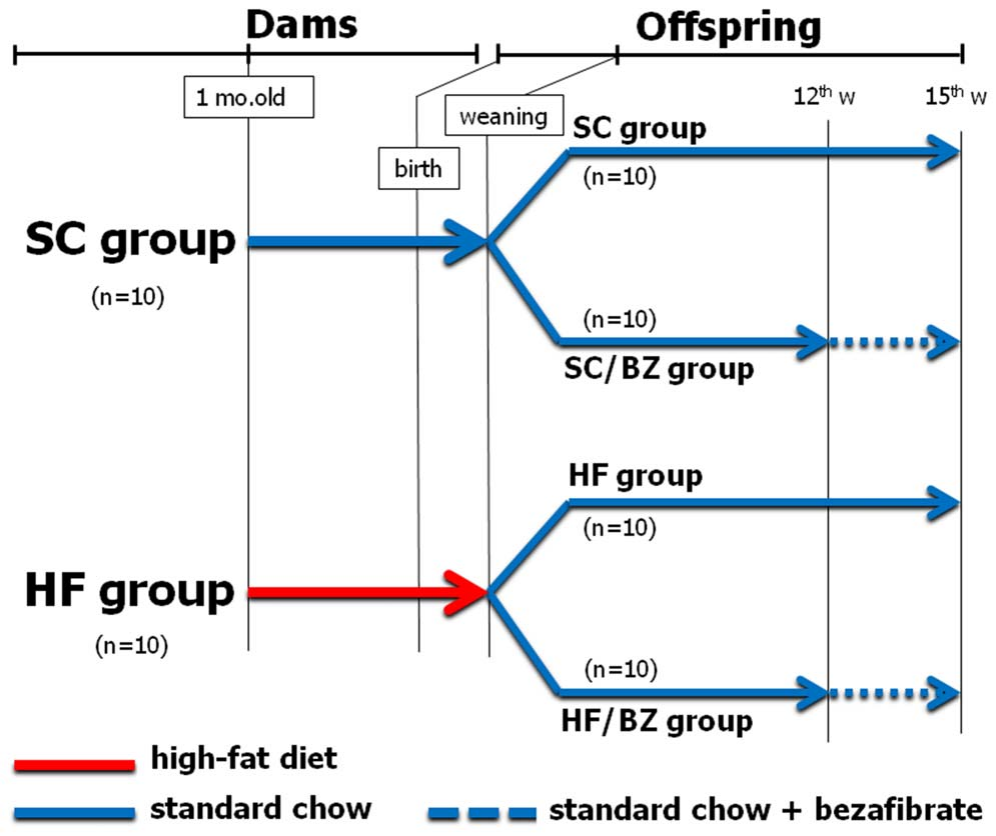
Formation of the experimental groups. Red arrow: period in which dams were fed a high-fat diet. Blue arrows: periods in which standard-chow was administered for dams or offspring. Dotted blue arrows: period of treatment with Bezafibrate. Abbreviations: Standard-chow group (SC); high-fat group (HF); standard-chow group with treatment with Bezafibrate (SC/BZ); high-fat group with treatment with Bezafibrate (HF/BZ); months old (mo. old); week (w).

Animals were maintained under controlled conditions (20±2°C, humidity 60±10% and 12 h/12 h dark/light cycle) and had free access to food and water. Four-week-old females were randomly divided into two nutritional groups (n = 10), standard-chow (SC; 10% of energy from lipids) or high-fat diet (HF; 49% of energy from lipids), and were fed these diets for 8 weeks. Food intake of dams was measured by Compulse v 2.7.13 (Harvard/Panlab, Barcelona, Spain) two weeks before mating, and body mass (BM) was measured weekly. At 12 weeks old, females were mated with breeding males, and during gestation and lactation, the females continued receiving the same diets. Dams received experimental diets during 8 weeks pre-mating in order to assure that they were overweighed at the time of conception. At weaning, male offspring were subdivided into four groups (n = 10), to include BZ treatment: SC, SC/BZ, HF, and HF/BZ (according to Figure 1).

Treatment with BZ started at 12 weeks of age and was maintained for three weeks. Food intake of offspring was measured by Compulse two weeks before sacrifice, and body mass (BM) was measured weekly. Offspring from HF and SC dams were weaned onto the same diet, SC diet. Therefore, metabolic abnormalities observed in HF offspring could be attributed to diet-induced maternal obesity as animals received balanced diet at post-weaning period. All diets were supplemented with purified nutrients by PragSolucoes (Jau, Sao Paulo, Brazil) and were in accordance with the American Institute of Nutrition's recommendations (Table 1). BZ (Sigma-Aldrich Co., St. Louis, Mo., USA) was added to the diets at the dose of 100 mg/kg, which is a murine dose that can be compared to the clinical human dose of 10 mg/kg [19].

**Table 1:** Composition and energy content of the HF (based on AIN 93G), SC1 (AIN 93G) and SC2 (AIN 93M) diets.

	Diets		
Content	HF*	SC1* (AIN93G)	SC2* ^/^ ** (AIN93M)
Casein (g/kg)	230	190	140
Cornstarch (g/kg)	299.486	539.486	250.69
Sucrose (g/kg)	100	100	100
Soybean oil (g/kg)	70	70	40
Lard (g/kg)	200	0	0
Fibre (g/kg)	50	50	50
Vitamin mix (g/kg)*	10	10	10
Mineral mix (g/kg)*	35	35	35
L-cystin (g/kg)	3	3	1.8
Choline (g/kg)	2.5	2.5	2.5
Antioxidant (g/kg)	0.014	0.014	0.014
Total mass (g)	1000	1000	1000
Energy (kcal/kg)	4950	3950	3570
Carbohydrates (%)	32	64	75.7
Protein (%)	19	19	14.9
Lipids (%)	49	17	9.4

* Mineral and vitamin mixtures are in accordance with AIN93-G (HF and SC1) and AIN93-M (SC2).

** Bezafibrate has been added to the diet at the dose of 100 mg/kg.

### Oral glucose tolerance test (OGTT)

OGTT was performed at one week before mating in dams, and at the end of treatment in offspring. Mice were fasted for 6 h before glucose administration. Glucose (1 g/kg) was given orally at time 0; tail blood was collected at fasting (i.e., baseline) and then at 15, 30, 60 and 120 min after glucose loading for glucose determination (Glucometer Accu-Chek, Roche, SP, Brazil). The area under the curve (AUC; millimolar per minute) was calculated. The values of glucose concentration at time 0 were used to determine the fasting glycemia.

### Sacrifice and tissue extraction

Before sacrifice, animals were deprived of food for 6 h and then deeply anaesthetized (150 mg/kg of sodium pentobarbital, intraperitoneally). Blood samples were rapidly obtained by cardiac puncture, and plasma was separated by centrifugation (120× g for 20 min) at room temperature and stored individually at −20°C until assay. Both the liver and the fat pads (inguinal, retroperitoneal and epididymal fat pads) were carefully dissected, weighed and prepared for analysis. Portions of the liver and the epididymal fat pad were kept in a freshly prepared fixative solution (formaldehyde 4% w/v, 0.1 M phosphate buffer pH 7.2) during 48 h and prepared to microscopy. Additional portions of the liver and epididymal fat pad were rapidly frozen for molecular analysis by immunoblotting and RT-qPCR. Remaining liver was prepared for biochemical analysis, and the adiposity index calculated and determined as the ratio between the sum of the masses of all fat pads divided by the total body mass, represented as a percentage [20].

### Plasma analysis

The concentrations of plasma total triglycerides (TG) of offspring were measured by automatic spectrophotometer using a commercial kit (Bioclin System II, Quibasa, Belo Horizonte, MG, Brazil). In the offspring, plasma concentrations of adiponectin, IL-6, insulin, leptin and resistin were evaluated using the Milliplex mouse serum adipokine panel kit MMHMAG-44K-08 (for IL-6, insulin, leptin and resistin) and MADPK-71K-ADPN (for adiponectin) with Luminex xMAP equipment (Millipore, Billerica, MA, USA).

### Homeostasis model assessment of insulin resistance index (HOMA-IR)

Serum insulin levels were determined using the Milliplex mouse serum adipokine panel kit MMHMAG-44K-08 and the fasting serum glucose were obtained at the sacrifice. The HOMA-IR index was calculated as: (fasting serum glucose X fasting serum insulin/22.5) [21]

### Adipocyte microscopy

Epididymal adipose tissue samples that were fixed, were embedded in Paraplast Plus (Sigma-Aldrich, St. Louis, MO, USA), sectioned at 5 µm and stained with hematoxylin and eosin (HE). Ten non-consecutive random microscopic fields were analyzed per animal on a light microscope (Leica Microsystems GmbH, Wetzlar, Germany) and an Infinity 1-5c camera (Lumenera Co., Otawa, ON, Canada). The mean diameter of at least 50 adipocytes per animal was measured using Image Pro Plus software v7.01 (Media Cybernetics, Silver Spring, MD, USA). The numerical density per area of adipocyte was evaluated in consideration of the number of adipocytes in a frame of known area when they did not hit two consecutive lines of the system (forbidden lines). The system was produced with the STEPanizer web-based system (www.stepanizer.com) [22].

### Liver microscopy and biochemistry

Random fragments of the liver were prepared for light microscopy. Small pieces of liver were embedded in Paraplast Plus (Sigma-Aldrich Co., St Louis, Mo., USA), sectioned at 5 µm and then stained with HE. Several slices were cut per fragment, and five microscopic fields per animal were analyzed at random. Digital images (same system described) and a test system made up of 36 test points (P_T_) produced with the STEPanizer web-based system were used for the analysis. The volume density (Vv) was estimated by point counting for hepatocytes and steatosis [23]: Vv[structure] = Pp[structure]/P_T_, where Pp is the number of points that hit the structure. Then, the numerical density per area of hepatocyte binucleation (binucleation/mm^2^) was estimated. A total of 50 digital images were evaluated per group, and all binucleation within the frame were counted except for those that hit the “forbidden lines” [24].

Several fragments of the liver of each animal were frozen at −80°C for further biochemical analysis. The hepatic triglyceride levels were measured. Briefly, 50 mg of frozen liver tissue was placed in an ultrasonic processor with 1 ml of isopropanol. The homogenate was centrifuged at 2000 g, and 5 µl of the supernatant was used to measure TAG with a kit and an automated spectrophotometer (Bioclin System II, Quibasa Ltda., Belo Horizonte, Brazil) [23].

### Immunoblotting

For liver and WAT analyses, total proteins were extracted in homogenizing buffer with protease inhibitors. Next, the homogenates were centrifuged at 4°C, and the supernatants were collected. Equal quantities of total protein were resuspended in SDS-containing sample buffer, heated for 5 min at 100°C and separated by SDS/PAGE. After electrophoresis, the proteins were electroblotted onto a polyvinyldifluoride transfer membrane (Amersham Biosciences, Piscataway, N.J., USA). The efficiency of the transfer was visualized by Ponceau solution staining. The membrane was blocked by incubation with non-fat dry milk. Antibodies against sterol regulatory element binding protein-1c (SREBP-1c), PPAR*alpha*, PPAR*gamma*, adiponectin, glucose-6-phosphatase (G6Pase), Phosphoenolpyruvate carboxykinase (PEPCK) and *beta*-actin were purchased from Santa Cruz. Hepatic and WAT homogenates were incubated with various polyclonal antibodies (as specified in the results section). *Beta*-actin served as a loading control for proteins.

All protein expression was detected using an ECL (enhanced chemiluminescence) detection system (Amersham Bioscience). Signals were visualized by autoradiography and determined by quantitative analysis of digital images of gels using ImageJ software, version 1.44 (NIH, imagej.nih.gov/ij, USA). The integral absorbance values were measured.

### Liver RT-qPCR

Total RNA was extracted from approximately 50 mg of liver tissue using Trizol reagent (Invitrogen, CA, USA). RNA amount was determined using Nanovue (GE Life Sciences) spectroscopy, and 1 µg of RNA was treated with DNAse I (Invitrogen). Synthesis of the first strand cDNA was performed using Oligo (dT) primers for mRNA and Superscript III reverse-transcriptase (both Invitrogen). Quantitative real time PCR (RT-qPCR) was performed using a Biorad CFX96 cycler and the SYBR Green mix (Invitrogen). Primers for qPCR were designed using the Primer3 online software and are indicated in Table 2. Endogenous control TBP (TATA Box binding-protein) was used to normalize the expression of the selected genes. Efficiencies of RT-qPCR for the target gene and the endogenous control were approximately equal, and were calculated through dilution series of cDNA. Real Time PCR reactions were conducted as follows: after a pre-denaturation and polymerase-activation program (4 min at 95°C), forty-four cycles, each one consisting of 95°C for 10 s and 60°C for 15 s were followed by a melting curve program (60 to 95°C with heating rate of 0.1°C/s). Negative controls consisted of wells in which cDNA was substituted for deionized water. The relative expression ratio (RQ) of mRNA was calculated by the equation 2^−ΔΔCt^, in which −ΔCT expresses the difference between number of cycles (CT) of the target genes and the endogenous control.

**Table 2:** RT-qPCR primers and respective sequences.

Name	5-3′	Primers
(FAT)/CD36	FW	TGCATTTGCCAATGTCTAGC
(FAT)/CD36	RV	CCCTCCAGAATCCAGACAAC
CPT-1	FW	AAGGAATGCAGGTCCACATC
CPT-1	RV	CCAGGCTACAGTGGGACATT
PPAR*alpha*	FW	TCGAGGAAGGCACTACACCT
PPAR*alpha*	RV	TCTTCCCAAAGCTCCTTCAA
PPAR*gamma2*	FW	ACGATCTGCCTGAGGTCTGT
PPAR*gamma2*	RV	CATCGAGGACATCCAAGACA
SREBP1-c	FW	TCTGCCTTGATGAAGTGTGG
SREBP1-c	RV	AGCAGCCCCTAGAACAAACA
TBP	FW	CAGCCTTCCACCTTATGCTC
TBP	RV	TTGCTGCTGCTGTCTTTGTT

Abbreviations: Fatty acid translocase (FAT/CD36); Carnitine palmitoyltransferase I (CPT-1); Peroxisome proliferator activator receptor alpha (PPAR*alpha*), Peroxisome proliferator activator receptor gamma2 (PPAR*gamma2); Sterol regulatory element binding protein 1-c (SREBP1-c); Tata Box binding-protein (TBP).*

### Data analysis

Values are shown as the mean and the standard error of mean (SEM). In the cases where we could confirm homoscedasticity of variances, comparisons among groups were made by a *t*-test or ANOVA followed by Holm-Sidak post-hoc test. A *P*-value≤0.05 was considered statistically significant (GraphPad Prism version 6.01 for Windows).

## Results

### Dams (before mating period)

Dams had no difference in BM (17.3±0.2 g) at 1 month of age. The HF dams had a higher energetic intake (+6%, *P*<0.03) and a higher BM gain than the SC dams (+29%, *P* = 0.043) during the 8 weeks before mating. Moreover, the AUC of the OGTT (+17%, *P*<0.02) and the fasting glucose levels (+13%, *P*<0.02) were higher in the HF dams than in the SC dams. These parameters are shown in Table 3.

**Table 3:** Data from the dam groups.

Data	Dam groups		
	SC	HF	*P* value
Food intake (g/day/mouse)	2.75±0.227	2.34±0.186	ns
Energy intake (kcal/day/mouse)	10.88±0.227	11.57±0.185	0.03
Initial body mass (g)	17.38±0.385	17.25±0.311	ns
Final body mass (g)	20.33±0.159	21.36±0.263	0.014
Body mass gain (g)	3.52±0.470	4.56±0.221	0.043
Fasting glucose (mg/dl)	101.5±4.35	114.8±1.38	0.027
OGTT (AUC, a.u.)	17010.0±869.5	19950.0±526.8	0.02

Data are expressed as the mean ± SEM. Differences were tested using Student's *t*-test, and significance values are shown in the *P*-value column.

Abbreviations: arbitrary units (a.u.); area under the curve (AUC); high-fat diet (HF); Oral glucose tolerance test (OGTT); standard-chow (SC).

### Offspring

#### Food and energy intake and BM evolution

**Figure 2:**
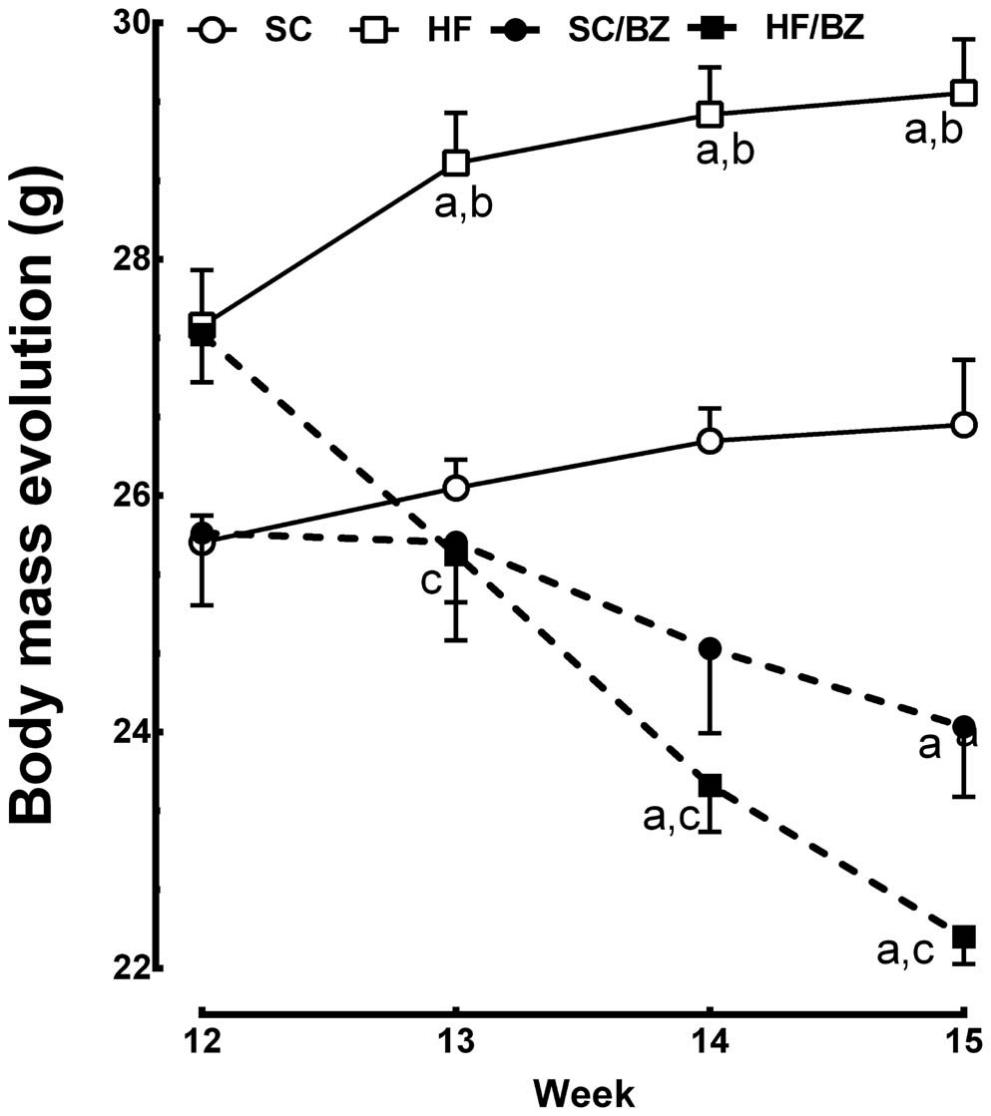
Body mass evolution over the treatment period (mean ± S.E.M., n = 10 per group). In signaled cases, *P*<0.05 when compared with SC [a], with SC/BZ [b], with HF [c] (one-way ANOVA and Holm-Sidak post-hoc test). Values are the. Abbreviations: Standard-chow for dams and offspring (SC); standard-chow for dams and offspring with treatment with Bezafibrate (SC/BZ); high-fat diet for dams and standard-chow for offspring (HF); high-fat diet for dams and standard-chow for offspring with treatment with Bezafibrate (HF/BZ).

The HF offspring had a higher food intake than the SC offspring (+21%, *P* = 0.0015). The SC/BZ and HF/BZ offspring ingested 17% and 14% less food than the SC and HF offspring, respectively (*P* = 0.0064). In regards to BM, there were no differences in the BM of the offspring up to 12 weeks of age, but after 13 weeks of age, the HF offspring were heavier than the SC offspring, and at the end of experiment, the HF offspring were 10% heavier than the SC offspring (*P* = 0.0024). Both groups of drug-treated offspring showed a reduced BM, and at the end of experiment, the SC/BZ and HF/BZ groups were 10% and 24% lighter, respectively, than their counterparts (*P* = 0.0061 *and P<0.0001*). Table 4 and Figure 2 show these findings.

**Table 4:** Data from the offspring groups.

	Offspring groups			
Data	SC	SC/BZ	HF	HF/BZ
Final body mass (g)	26.61±0.552	24.12±0.586 [a]	29.31±0.418 [a]	22.27±0.232 [a,c]
Food intake (g/day per mouse)	2.47±0.080	2.05±0.092 [a]	3.00±0.081 [a]	2.58±0.084 [c]
Energy intake (kcal/day per mouse)	8.83±0.267	7.30±0.424 [a]	10.71±0.286 [a]	9.23±0.301 [c]
Fasting glucose (mg/dl)	110.1±3.46	110.4±2.99	136.2±5.04 [a]	87.2±3.51 [a,b,c]
OGTT (AUC, a.u.)	14519.0±488.5	14924.0±267.7	18338.0±766.6 [a]	13260.0±908.4 [c]
Plasmatic triglycerides (mg/dl)	54.33±2.582	52.63±1.502	66.83±3.817 [a]	49.36±1.567 [a,b,c]
Adiponectin (10^7^ pg/ml)	1.24±0.053	1.82±0.086 [a]	0.885±0.054 [a]	1.80±0.121 [a,c]
IL-6 (pg/ml)	32.83±6.703	23.73±6.218	146.81±15.360 [a]	73.48±10.144 [b, c]
Insulin (pg/ml)	209.11±38.190	220.83±20.682	421.22±85.061 [a]	263.20±26.612 [c]
Leptin (pg/ml)	277.62±23.761	276.76±29.883	376.42±18.963 [a]	187.41±24.621 [c]
Resistin (pg/ml)	1594.62±150.423	1342.23±89.334	2721.15±109.232 [a]	2136.23±80.334 [a,b,c]
HOMA-IR	1.51±0.3107	1.488±0.1328	3.96±0.9718 [a,b]	1.38±0.2042 [c]

Data are expressed as the mean ± SEM. In signaled cases (one-way ANOVA and Holm-Sydak post-hoc test), *P*<0.05 when compared with the SC offspring [a], with SC/BZ offspring [b], and with HF offspring [c], n = 10 per group.

Abbreviations: arbitrary units (a.u.); area under the curve (AUC); homeostasis model assessment of insulin resistance index (HOMA-IR); interleukin-6 (IL-6); Oral glucose tolerance test (OGTT);

Groups: standard-chow for dams and offspring (SC); standard-chow for dams and offspring with treatment with Bezafibrate (SC/BZ); high-fat diet for dams and standard-chow for offspring (HF); high-fat diet for dams and standard-chow for offspring with treatment with Bezafibrate (HF/BZ).

#### Carbohydrate metabolism

The HF offspring had higher fasting glucose values than the SC offspring (+23%, *P* = 0.0009), while the HF/BZ offspring showed a decrease in fasting glucose levels in comparison with the HF (−36%, *P<0.0001*), the SC (−23%, *P* = 0.002) and the SC/BZ (−21%, *P* = 0.0017) offspring. Additionally, as shown in table 4, impairment in glucose tolerance in the HF offspring in comparison with the SC offspring (+26% for the AUC, *P* = 0.0041) was observed, which was minimized by the BZ treatment in the HF/BZ offspring (−27% for the AUC, *P* = 0.0003). Moreover, also presented in table 4, the HOMA-IR indicates that the HF offspring presented insulin resistance when compared to the SC offspring (+162%, *P<0.05*) and the treatment with BZ improved this parameter in the HF/BZ offspring (−65%, *P<0.05*).

#### Plasma analysis

Plasma TG levels were affected by maternal obesity as the HF offspring had a higher concentration of TG than the SC offspring (+23%, *P*<0.0001). However, the HF/BZ offspring showed an improvement in this parameter in comparison to the HF offspring, with a consistent decrease of 26% (*P*<0.0001). Furthermore, the plasma TG levels in the HF/BZ offspring were lower than those in the SC (−10%, *P* = 0.0008) and the SC/BZ (−6%, *P* = 0.011) offspring (Table 4).

Adiponectin, IL-6, insulin, leptin and resistin levels in offspring were also affected by diet-induced maternal obesity, but BZ improved all these parameters, especially in the HF/BZ group, as shown in table 4.

#### Liver mass and hepatic TG levels

The HF offspring had greater liver mass than the SC offspring (+19%, *P* = 0.008), and both groups of treated offspring had increased liver masses in relation to their controls (+103% for SC/BZ; +53% for HF/BZ, *P*<0.0001). Despite presenting larger liver masses, both treated offspring groups presented reduced hepatic triglycerides: the SC/BZ group showed lower concentrations than the SC group (−15%, *P* = 0.003), and the HF/BZ group had a reduction of 16% when compared to the untreated HF group (*P* = 0.0006). Conversely, the HF offspring had a higher hepatic triglyceride concentration than the SC offspring (+21%, *P* = 0.0003). All of these findings are described in table 5.

**Table 5:** Liver and white adipose tissue parameters.

Liver	Offspring groups			
	SC	SC/BZ	HF	HF/BZ
Mass (g)	0.3695±0.0271	0.7525±0.2765 [a]	0.4054±0.0499 [a]	0.6238±0.0629 [a,c]
Hepatic triglycerides (mg/dl)	110.5±3.06	94.5±1.68 [a]	133.9±5.97 [a]	112.4±2.55 [b,c]
Steatosis (%)	10.1±0.87	5.0±0.638 [a]	24.6±2.36 [a]	3.5±0.57 [a,c]
Binucleation (1/µm^2^)	231.3±18.9	136.3±11.9 [a]	338.0±32.9 [a]	231,3±5.8 [c]
**Adipose tissue**				
Epididymal/subcutaneous ratio	1.32±0.074	1.23±0.062	1.73±0.076 [a]	1.09±0.111 [c]
Adiposity index (%)	2.3±0.03	2.2±0.04	2.5±0.06 [a]	2.2±0.02 [c]
Adipocyte size (µm)	31.3±0.8	26.1±1.4 [a]	38.9±1.2 [a]	26.4±0.7 [a,c]
Adipocyte density (1/mm^2^)	514.8±29.4	678.3±38.1 [a]	351.5±16.5 [a]	623.5±23.4 [a,c]

Data are expressed as the mean ± SEM. In signaled cases (one-way ANOVA and Holm-Sydak post-hoc test), *P*<0.05 when compared with the SC offspring [a], with SC/BZ offspring [b], and with HF offspring [c], n = 10 per group.

Groups: standard-chow for dams and offspring (SC); standard-chow for dams and offspring with treatment with Bezafibrate (SC/BZ); high-fat diet for dams and standard-chow for offspring (HF); high-fat diet for dams and standard-chow for offspring with treatment with Bezafibrate (HF/BZ).

#### Liver steatosis and binucleation

The presence of liver steatosis was confirmed in the HF group, and its improvement was shown in the HF/BZ group using stereological quantification. Fewer lipid droplets were observed in the livers of the SC group (11.69±1.3%), which was considered normal, while the SC/BZ group exhibited a sharp decrease (−49%, *P* = 0.014) in liver lipid droplet levels. Although steatosis was substantial in the HF group (+143%, *P*<0.0001), the BZ treatment promoted a significant decrease in the HF/BZ group (−85%, *P*<0.0001). These findings are depicted in table 5.

Numerical density of binucleation showed that the HF group had higher profiles of binucleation than the SC group (+57%, *P* = 0.004, table 4), while the HF/BZ group showed a decrease in the binucleation profile in relation to the untreated HF group (−31%, *P* = 0.004, table 5).

#### Fat pad mass and adipose index

The HF mice showed an increase in epididymal (+24%, *P* = 0.0008) and retroperitoneal (+26%, *P* = 0.007) fat pad mass and a decrease in inguinal fat pad mass (−20%, *P* = 0.0006) compared with the SC mice. The SC/BZ group presented a decrease (−16%, *P* = 0.014) in epididymal fat pad mass compared with the SC group, whereas, the HF/BZ group presented a decrease in epididymal and retroperitoneal fat pad mass compared with untreated HF group (−43%, −44%, respectively, *P*<0.0001). The epididymal/inguinal fat pad mass ratio was higher for the HF group than for the SC group (+31%, *P* = 0.016), and treatment with BZ reversed this ratio in the HF/BZ group (−37%, *P*<0.0001). Similarly, the adipose index was elevated for the HF group when compared to the SC group (+8%, P = 0.004), and the HF/BZ group showed a decrease in the adipose index (−13%, *P*<0.0001) when compared with the untreated HF group (Table 5).

#### Adipocyte diameter

The adipocytes of the HF mice were considerably enlarged in comparison to those of the SC mice (+24%, *P* = 0.0004). The SC/BZ and HF/BZ mice had adipocytes of a decreased size (−16%, *P* = 0.009; −34%, *P*<0.0001, respectively) in comparison with their controls. The numerical density of adipocytes per unit area in the HF group was smaller than in the SC group (−31%, *P* = 0.0005); however, the SC/BZ group showed an elevated numerical density of adipocytes per unit area when compared to the SC group (+31%, *P* = 0.0005). The HF/BZ group showed similar results, with the numerical density of adipocytes per unit area of adipocytes per unit area 77% higher than in the untreated HF group (*P*<0.0001) and 21% higher than in the SC group (*P = 0.02*) (table 5).

#### Adipocyte diameter distribution (ADD)

**Figure 3:**
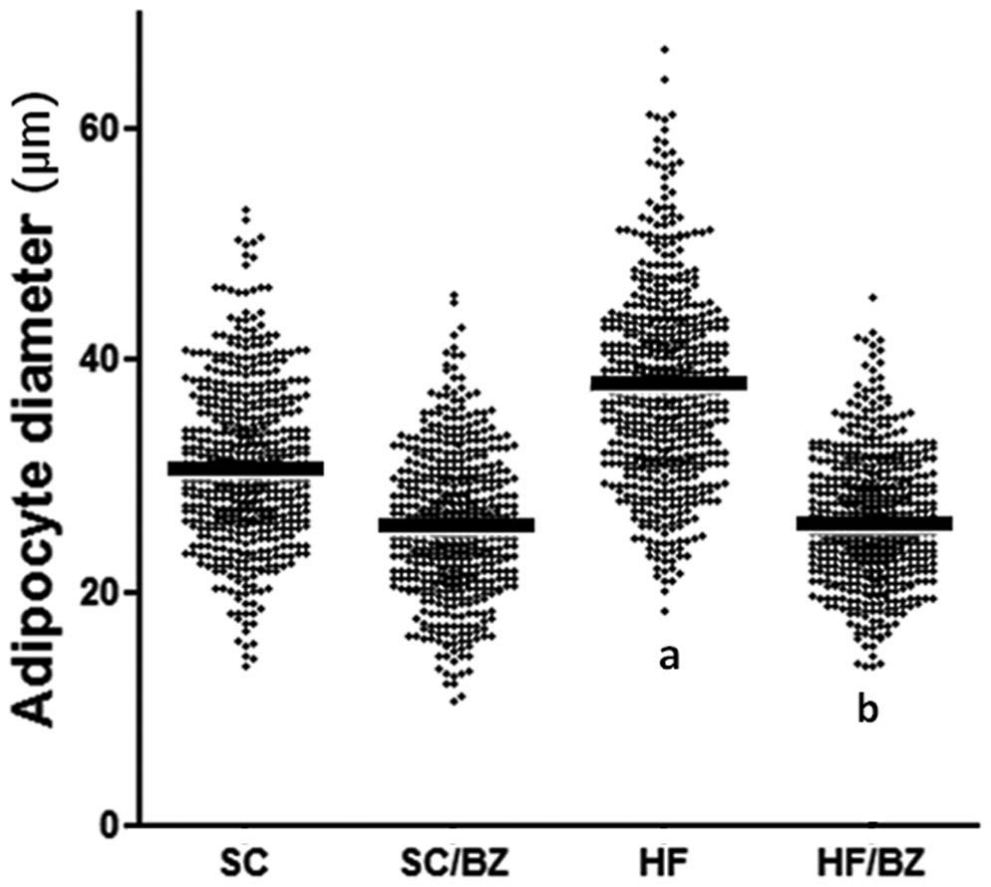
Adipocyte diameter distribution with medians (black bars). In signaled cases, *P*<0.05 when compared with SC [a], and with HF [b] (one-way ANOVA and Holm-Sidak post-hoc test). Abbreviations: Standard-chow for dams and offspring (SC); standard-chow for dams and offspring with treatment with Bezafibrate (SC/BZ); high-fat diet for dams and standard-chow for offspring (HF); high-fat diet for dams and standard-chow for offspring with treatment with Bezafibrate (HF/BZ).

When ADD was performed, the HF group had higher numbers of large adipocytes than the SC group (*P*<0.0001), most likely due to maternal obesity. However, the HF/BZ group had higher amounts of small adipocytes than the HF group (*P*<0.0001), with similar values to the SC and SC/BZ groups. The ADD can be observed in figure 3.

#### Liver immunoblotting

**Figure 4:**
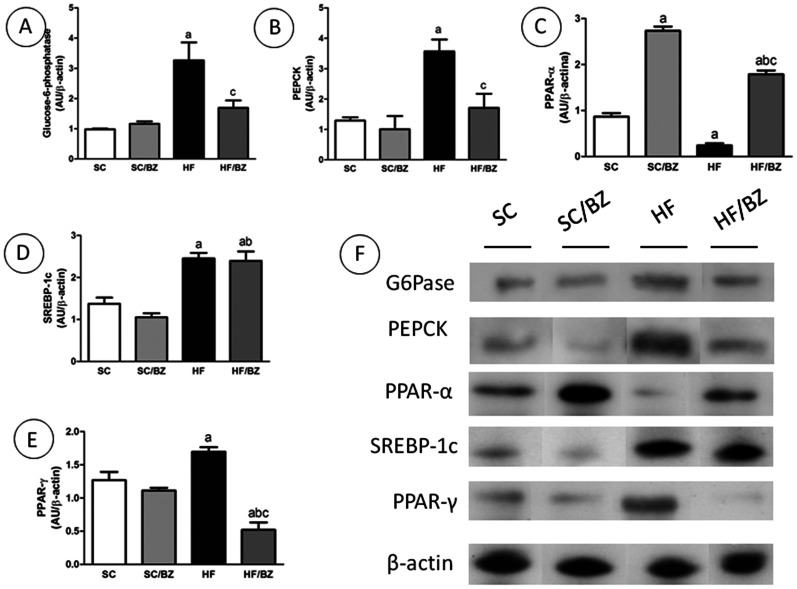
Liver immunoblot corrected by beta-actin expression (mean ± S.E.M., n = 5 per group). (A) G6Pase expression; (B) PEPCK expression; (C) PPAR*alpha* expression; (D) SREBP-1c expression; (E) PPAR*gamma* expression; (F) representative immunoblot with bands corresponding to the offspring (expressed in arbitrary units. In the signaled cases, *P*<0.05 when compared with SC [a], with SC/BZ [b], and with HF [c] (one-way ANOVA and post-hoc Holm-Sidak test). Abbreviations: Standard-chow for dams and offspring (SC); standard-chow for dams and offspring with treatment with Bezafibrate (SC/BZ); high-fat diet for dams and standard-chow for offspring (HF); high-fat diet for dams and standard-chow for offspring with treatment with Bezafibrate (HF/BZ); peroxisome proliferator-activated receptor *alpha* (PPAR*alpha*); peroxisome proliferator-activated receptor *gamma* (PPAR*gamma*); glucose-6-phosphatase (G6Pase), phosphoenolpyruvate carboxykinase (PEPCK); arbitrary units (AU).

In order to verify the hepatic IR, G6Pase and PEPCK enzymes were assessed by immunoblotting. The HF offspring presented an elevated expression of G6Pase (*P* = 0.001) while the HF/BZ offspring showed a decreased expression of the same enzyme (*P*<0.05) (Figure 4A). Similar results were found in relation to PEPCK in that the HF offspring presented an elevated expression of PEPCK (*P*<0.05) and the HF/BZ offspring, a decrease expression of the same enzyme (*P*<0.05) (Figure 4B). In consideration of *beta*-*oxidation* and lipogenesis, PPAR*alpha* expression was decreased in the HF group when compared to the SC group (*P*<0.0001) and increased in both BZ-treated groups in comparison with their counterparts (*P*<0.0001); however, the SC/BZ group showed elevated expression of this TF in comparison with the HF/BZ group (*P*<0.0001) (Figure 4C). In contrast, we observed that SREBP-1c expression was higher in the HF group than in the SC group (*P* = 0.0007); nevertheless, treatment with BZ did not affect the expression of this TF (Figure 4D). Although PPAR*gamma* expression in the HF group was also elevated in comparison to the SC group (*P* = 0.01), it was decreased in the HF/BZ group in relation to the untreated HF group (*P*<0.0001) (Figure 4E).

**Figure 5:**
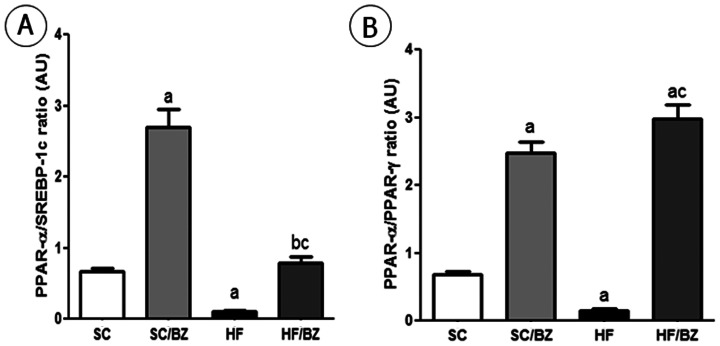
Liver immunoblot corrected by beta-actin expression (mean ± S.E.M., n = 5 per group). (A) PPAR*alpha*/SREBP-1c ratio; (B) PPAR*alpha*/PPAR*gamma* ratio (expressed in arbitrary units, AU). In the signaled cases, *P*<0.05 when compared with SC [a], with SC/BZ [b], and with HF [c] (one-way ANOVA and post-hoc Holm-Sidak test). Abbreviations: Standard-chow for dams and offspring (SC); standard-chow for dams and offspring with treatment with Bezafibrate (SC/BZ); high-fat diet for dams and standard-chow for offspring (HF); high-fat diet for dams and standard-chow for offspring with treatment with Bezafibrate (HF/BZ); sterol regulatory element binding protein-1c (SREBP-1c); peroxisome proliferator-activated receptor *alpha* (PPAR*alpha*); peroxisome proliferator-activated receptor *gamma* (PPAR*gamma*); arbitrary units (AU).

Considering the results above, the PPAR*alpha*/SREBP-1c ratio was lower in the HF group compared to the SC group (*P* = 0.0176), higher in the SC/BZ group compared to the SC group (*P*<0.0001) and higher in the HF/BZ group compared to the untreated HF group (*P* = 0.008) (Figure 5A). Likewise, the PPAR*alpha*/PPAR*gamma* ratio was higher in the SC/BZ group compared to the SC group (*P*<0.0001) and in the HF/BZ group compared to the HF group (*P*<0.0001). Moreover, the PPAR*alpha*/PPAR*gamma* ratio was lower in the HF group compared to the SC group (*P* = 0.01) (Figure 5B).

#### WAT immunoblotting

**Figure 6:**
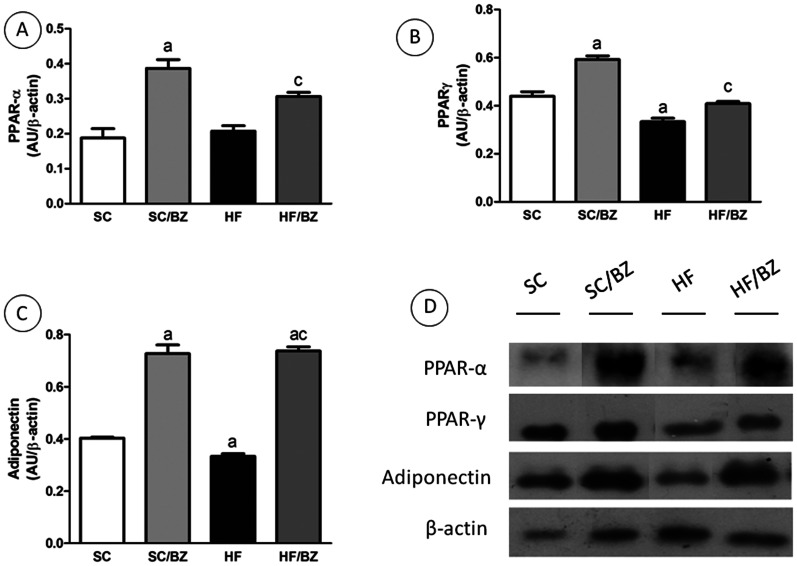
White adipose tissue immunoblot corrected by beta-actin expression (mean ± S.E.M., n = 5 per group). (A) PPAR*alpha* expression; (B) PPAR*gamma* expression; (C) Adiponectin expression; (D) representative immunoblot with bands corresponding to the offspring (expressed in arbitrary units, AU). In the signaled cases, *P*<0.05 when compared with SC [a], with SC/BZ [b], and with HF [c] (one-way ANOVA and post-hoc Holm-Sidak test). Abbreviations: Standard-chow for dams and offspring (SC); standard-chow for dams and offspring with treatment with Bezafibrate (SC/BZ); high-fat diet for dams and standard-chow for offspring (HF); high-fat diet for dams and standard-chow for offspring with treatment with Bezafibrate (HF/BZ); peroxisome proliferator-activated receptor *alpha* (PPAR*alpha*); peroxisome proliferator-activated receptor *gamma* (PPAR*gamma*); arbitrary units (AU).

There is no significant difference between the SC offspring and the HF offspring in relation to PPAR*alpha* expression, but both treated group presented an elevation in the expression of this TF (*P* = 0.001 for the SC/BZ group in comparison with the SC group and *P*<0.05 for the HF/BZ group in comparison with the HF group, figure 6A). *PPARgamma* expression in adipose tissue was lower in the HF group than in the SC group (*P* = 0.003), and both drug-treated groups presented an elevation in the expression of this TF (*P* = 0.0004 for the SC/BZ group in comparison with the SC group and *P* = 0.0147 for the HF/BZ group in comparison with the HF group, figure 6B). Similarly, adiponectin expression was decreased in the HF group (*P* = 0.03), and treatment with BZ restored the expression in both treated groups (*P*<0.0001) (Figure 6C).

#### Liver RT-qPCR

**Figure 7:**
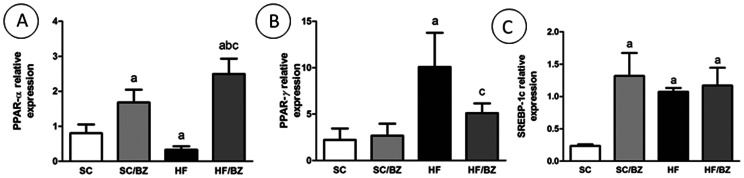
PPAR*alpha* (A), PPAR*gamma* (B) and SREBP-1c (C) mRNA levels. Endogenous control TBP (TATA Box binding-protein) was used to normalize the expression of the selected genes. In the signaled cases, *P*<0.05 when compared with SC [a], with SC/BZ [b], and with HF [c] (one-way ANOVA and post-hoc Holm-Sidak test). Abbreviations: Standard-chow for dams and offspring (SC); standard-chow for dams and offspring with treatment with Bezafibrate (SC/BZ); high-fat diet for dams and standard-chow for offspring (HF); high-fat diet for dams and standard-chow for offspring with treatment with Bezafibrate (HF/BZ); peroxisome proliferator-activated receptor *alpha* (PPAR*alpha*); peroxisome proliferator-activated receptor *gamma* (PPAR*gamma*); Sterol regulating element binding protein (SREBP-1c).

**Figure 8:**
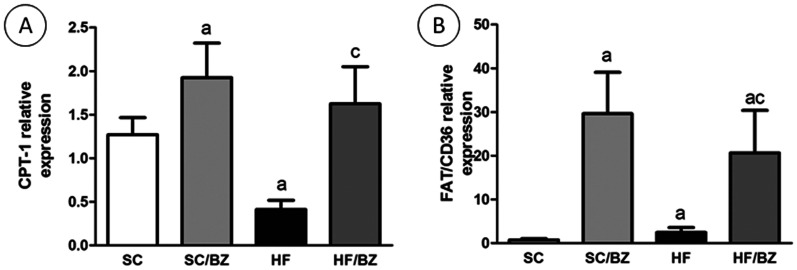
CPT-1 (A) and FAT/CD36 (B) mRNA levels. Endogenous control TBP (TATA Box binding-protein) was used to normalize the expression of the selected genes. In the signaled cases, *P*<0.05 when compared with SC [a], with SC/BZ [b], and with HF [c] (one-way ANOVA and post-hoc Holm-Sidak test). Abbreviations: Standard-chow for dams and offspring (SC); standard-chow for dams and offspring with treatment with Bezafibrate (SC/BZ); high-fat diet for dams and standard-chow for offspring (HF); high-fat diet for dams and standard-chow for offspring with treatment with Bezafibrate (HF/BZ); Carnitine palmitoyltransferase I (CPT-1); Fat acid translocase (FAT/CD36).

Considering the relative mRNA expression of PPAR*alpha* and its target gene, CPT-1, it was found that both treated groups presented an elevation of both mRNAs (*P*<0.001) and, inversely, the HF group presented a decreased expression of these mRNAs (*P*<0.05 for PPAR*alpha* mRNA and *P*<0.01 for CPT-1 mRNA). In addition to these findings, relative mRNA expression of PPAR*gamma* was increased in the HF group (*P*<0.001) while the HF/BZ presented a decreased expression (*P*<0.05). Interestingly, CD36, a target gene of PPAR*gamma*, presented an elevated expression in both treated groups (*P*<0.001) and the HF group also presented a slight elevation of this protein (*P*<0.05). Relative mRNA expression of SREBP-1c was elevated in both treated groups and also in the HF group (P<0.001) (Figures 7 and 8).

**Figure 9:**
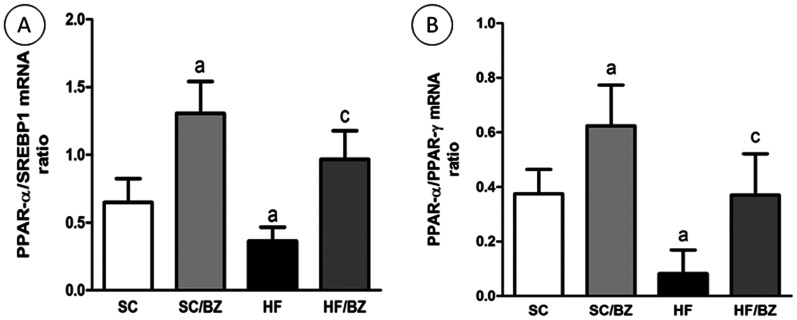
PPAR*alpha*/SREBP1-c mRNA ratio (A) and PPAR*alpha*/PPAR*gamma* mRNA ratio (B). In the signaled cases, *P*<0.05 when compared with SC [a], with SC/BZ [b], and with HF [c] (one-way ANOVA and post-hoc Holm-Sidak test). Abbreviations: Standard-chow for dams and offspring (SC); standard-chow for dams and offspring with treatment with Bezafibrate (SC/BZ); high-fat diet for dams and standard-chow for offspring (HF); high-fat diet for dams and standard-chow for offspring with treatment with Bezafibrate (HF/BZ); peroxisome proliferator-activated receptor *alpha* (PPAR*alpha*); peroxisome proliferator-activated receptor *gamma* (PPAR*gamma*).

Hence, the PPAR*alpha*/SREBP-1c and the PPAR*alpha*/PPAR*gamma* ratios were higher in both treated groups when compared to the SC group and to the HF group (*P*<0.001) and the HF group also presented a decreased ratio in comparison to the SC group (*P*<0.05) (Figure 9).

## Discussion

Diet-induced obesity in dams during pre-mating, gestation and lactation periods produced offspring with noticeable BM gain, high levels of plasma and hepatic TGs, high levels of pro-inflammatory and low levels of anti-inflammatory adipokines, impairment of glucose metabolism, abnormal fat pad mass distribution, higher numbers of larger adipocytes, hepatic steatosis, high mRNA expression of lipogenic proteins and its target genes concomitant to decreased expression of PPAR*alpha* and CPT-1 in liver, and diminished expression of PPAR*gamma* and adiponectin in WAT. All these findings could be accounted for by diet-induced maternal obesity once all offspring received a balanced diet at weaning instead of the same diet as their mothers.

Treatment with BZ ameliorated the hepatic defects generated by maternal obesity as well as WAT remodeling, with improvement in the structural, biochemical and molecular characteristics of the animals' livers and epididymal WAT. These benefits can be attributed to drug administration and the negative energy balance observed in treated animals since such improvements were not exhibited by animals exposed to diet-induced maternal obesity without being treated.

HF offspring had hyperphagia, an expected behavior accounted for by hypotalamic modifications owing to excessive maternal energy intake, leading to obesity in adulthood [25], [26]. BZ yielded negative energetic balance with decreased food intake in both treated offspring groups and this observation agrees with the reduced body mass observed in treated offspring compared to their counterparts [27].

Diet-induced maternal obesity triggers increased non-esterified fatty acids (NEFA), triglycerides, glucose and inflammatory cytokine influx to the fetus, which provoke dyslipidemia and glucose intolerance in the HF offspring [3], [26]. Moreover, the HF offspring also showed higher levels of plasma insulin and leptin than the SC offspring. Increased adiposity index correlates with elevated circulating levels of leptin, which in the pancreas act as a regulator of insulin secretion in a so called “adipose-insular axis”. Under normal physiological conditions, insulin stimulates adipogenesis and leptin production, whereas leptin inhibits the production of insulin by pancreatic beta cells. However, as the individual put on weight, adipose tissue stores increase and leptin reaches supraphysiological levels and loses its capacity to suppress insulin production. As a result, hyperleptinemia is followed by hyperinsulinemia, which leads to hyperglycemia in the long term [28]. Furthermore, it is widely known that normal levels of insulin inhibit G6Pase and PEPCK gene transcription and overexpression of these enzymes is a consequence of IR [29]. In this study, it was demonstrated that the HF offspring presented an elevated expression of both enzymes that characterizes IR. On the other hand, treatment with BZ reduced the expression of theses enzymes in both treated groups, pointing the effects of BZ on improvement of insulin sensibility.

Activation of PPAR*alpha* by BZ is widely known as an important step to produce its hypolipidemic effects [30]. Reducing adiposity index and modifying WAT's secretion profile, BZ yields normalization of leptin levels and restoration of adequate insulin levels. This explanation agrees with glucose metabolism improvement observed in the drug-treated offspring from diet-induced obese dams.

The HF offspring's WAT showed elevated numbers of larger adipocytes in comparison with the SC offspring, complying with expansion of visceral WAT and hypertrophy of adipocytes. Thus, these animals also showed altered metabolism of WAT, showing increased release of pro-inflammatory adipokines such as IL-6, resistin and TNF-*alpha* and decreased adiponectin plasma levels as observed in previous studies [5], [31]. Treatment with BZ has beneficial effects attenuating the pro-inflammatory adipokine profile in the HF/BZ offspring. Several works have demonstrated that activation of the PPAR isoforms could change the obesity-induced inflammatory state [18], [32]. PPAR*alpha* activation serves as a negative regulator of the inflammatory process by antagonizing the activity of tissue factor pathways linked to inflammation such as the nuclear factor (NF)-*kappa*B [33].

PPAR*gamma* activation in WAT is responsible for decreased production of adipokines that cause IR, such as TNF*alpha* and resistin [34]. Additionally, PPAR*gamma* activation causes apoptosis of mature and large adipocytes and increases the population of small adipocytes, which are more insulin sensitive [35]. These findings are in accordance with results found here in drug-treated offspring such as increased expression of PPAR*gamma* in WAT, the increase in the number of adipocytes per area and in the population of small adipocytes, as well as decreased adipocyte diameter, indicating an improvement of morphology and consequently metabolism of this tissue. These data indicate that BZ is a potent drug that can be used to reverse the WAT inflammatory state through PPAR*gamma* activation. Besides, PPAR*alpha* also has an important role in mitochondrial fatty acid oxidation in adipocytes even with lower PPAR*alpha* mRNA expression level in adipocytes than in the liver. Furthermore, activation of this TF also leads to adipocytes differentiation, suggesting that PPAR*alpha*-specific adipogenic pathways exist, although the effect of PPAR*alpha* seems to be partially shared with that of PPAR*gamma* [36]. Our results show that both drug-treated groups presented an elevation in the expression of PPAR*alpha* in WAT. Interestingly, there is no difference between the SC offspring and the HF offspring, but it is important to consider and highlight that the HF offspring were weaned onto a balanced diet.

Adiponectin down-regulation in obesity is a key factor in the development of NAFLD because it is closely linked to IR and diabetes [18], [20]. Likewise plasmatic levels, WAT expression of adiponectin was lower in the HF offspring than in the SC offspring. Both conditions were reversed by treatment with BZ. Furthermore, adiponectin is a direct target of regulation by PPAR*gamma*, whose expression was also increased in WAT from our treated animals, corroborating our results [37]. When treated with Pioglitazone, a PPAR*gamma* agonist, 3T3-L1 adipocytes presented enhanced insulin sensitivity through an upregulation of adiponectin receptor 2 (AdipoR2) and adiponectin, a novel pathway that might be addressed [38].

The high hepatic TG levels found in the HF offspring was alleviated by treatment in HF/BZ offspring. These data correlate with hepatic steatosis, confirming the effects of diet-induced maternal obesity in the development of NAFLD [3]. Moreover, higher level of hepatocyte binucleation in the HF offspring than in the SC offspring indicates an unsuccessful attempt to recover from liver damage [12]. Targeting PPARs with BZ reduced hepatic steatosis as well as binucleation rate, leading to improvement in hepatic structure.

PPAR*alpha* activation by fibrates in rodents induces hepatic peroxisomal proliferation and hepatomegaly [39]. In fact, the dose administered caused an increase in liver mass in both treated groups; however, this effect is lost in humans, even when fibrates maintain PPAR*alpha* activation. It has been proposed that PPAR*alpha* is more highly expressed in the hepatic tissue in rodents than in humans [40]. However, the role of PPAR*alpha* as the master regulator of hepatic lipid metabolism is well conserved between these species [41].

TG accumulation in the liver is a result of increased hepatic fatty acid synthesis and/or decreased *beta*-oxidation [42]. PPARs target differently hepatic *beta*-oxidation and lipogenesis [42] and in order to gain insight into molecular mechanism involved into these processes, evaluation of the expression of some proteins as well as mRNA expression are relevant. PPAR*alpha* target genes encode enzymes involved with mitochondrial *beta*-oxidation [43], by which PPAR*alpha* has a role in energy homeostasis, contributing greatly to energy production via oxidative phosphorylation generating ATP [42]. Our findings showed that both drug-treated groups exhibited high levels of PPAR*alpha* expression and that conversely, the HF offspring showed lower levels of PPAR*alpha* expression after exposure to diet-induced maternal obesity. The expression of PPAR*alpha* correlates inversely with hepatic steatosis degree. CPT-1, a target gene of PPAR*alpha*, is the mitochondrial gateway for fatty acid entry into the mitochondrial matrix, being the master regulator of the hepatic mitochondrial *beta*-oxidation [42], [44], and it was reported that patients with NAFLD presents a decreased expression of CPT-1 gene [45]. Our findings showed that BZ could restore the expression of CPT-1 in both treated groups, ameliorating the hepatic steatosis caused by maternal diet-induced obesity.

Although PPAR*gamma* is expressed at low concentrations in hepatic tissue, patients with NAFLD/NASH exhibit significantly high levels [46]. Concomitantly, SREBP-1c is also found in high levels in patients with NAFLD, acting with PPAR*gamma* to favor lipogenesis and accumulation of triglycerides in hepatic tissue [3], [47]. According to our data, diet-induced maternal obesity led to an increase in both PPAR*gamma* and SREBP-1c in HF offspring, which justifies the steatosis found in this group. However, treatment with BZ decreased PPAR*gamma* expression in the liver of the HF/BZ offspring in comparison to the untreated HF offspring, reducing hepatic lipogenesis.

Fatty acid translocase (FAT)/CD36, a PPAR*gamma* target gene, is involved with long chain fatty acid (LCFAs) transport into mitochondria, correlating with oxidative capacity of the liver as long as CPT-1 is also present [44], [48]. Although (FAT)/CD36 does not play a significant role in fatty acid uptake in liver, it makes possible the initial metabolization of LCFA, consisting of shortening its chain to enable the fatty acid to enter the mitochondria through CPT-1 action (maximum of 20 carbons in the molecule) [48]. Although HF group presented enhanced expression of (FAT)/CD36 mRNA, those animals also had lower levels of CPT-1 mRNA. Hence, the acetyl-coA resulting from the shortening of LCFA that cannot enter *beta*-oxidation pathway and might be deviated to lipogenesis and/or ketone bodies formation [42]. Besides, excessive fatty acids could be stored as lipid droplets. Conversely, the increased mRNA levels of CD36 in treated groups reinforce the activation of the three PPARs isoforms by BZ as PPAR*beta* activation increases the capacity of PPAR*gamma* to induce CD36 transcription [49]. A previous work showed that Bezafibrate (Pan-PPAR agonist) overcome Troglitazone (total PPAR*gamma* agonist) potential to induce CD36 [50]. In the present study, interplay between (FAT)/CD36 and CPT-1, both higher expressed in treated groups, guarantees the maximum oxidative activity within hepatic mitochondria, enhancing fatty acid metabolization and, thus, reducing hepatic steatosis.

The reduced PPAR*alpha*/SREBP-1c ratio observed in the untreated HF offspring indicates higher SREBP-1c activation with lower PPAR*alpha* expression, agreeing with a previous report [51] that showed higher SREBP-1c/PPAR*alpha* ratio in obese patients. Although BZ did not modify SREBP-1c expression levels in the HF group, the activation of PPAR*alpha* in the treated groups is more significant than SREBP-1c activation, favoring *beta*-oxidation. Similarly, the PPAR*alpha*/PPAR*gamma* ratio was significantly decreased in the offspring from diet-induced obese dams and increased in the treated offspring.

The literature emphasizes that both PPAR*gamma* and SREBP-1c are essential to trigger hepatic lipogenesis [51]. However, SREBP-1c seems to be an auxiliary of PPAR*gamma* [52], which emerges as the master regulator of hepatic lipogenesis. In this way, SREBP-1c deletion in mice results in 50% less fatty acid synthesis [53], whereas mice deficient in liver-specific PPAR*gamma* are completely protected against steatosis development [54]. In the present study, treatment with BZ did not modify SREBP-1c expression in the HF/BZ offspring when compared to the HF offspring. Despite unchanged expression of SREBP-1c mRNA after treatment with BZ, levels of PPAR*gamma* mRNA were considerably reduced, being crucial to the lower hepatic triglycerides and liver steatosis found in this treated group.

Additionally, the relative abundance of PPAR*alpha* in normal liver depicted by and increased PPAR*alpha*/PPAR*gamma* ratio in treated groups, causes this receptor to act as a regulator of fatty acid catabolism, minimizing the need to store these lipids in hepatocytes. Therefore, PPAR*alpha* could protect the liver against PPAR*gamma* lipogenic activity [55].

All of these findings support the conclusion that BZ can ameliorate hepatic metabolic abnormalities because it causes an increase in *beta*-oxidation via PPAR*alpha* activation and rise in the target gene CPT-1 in association with a decrease in PPAR*gamma* expression and no change in SREBP-1c expression.

The observation that increased PPAR*alpha*/PPAR*gamma* ratio counters hepatic lipogenesis, even with a lack of effect upon SREBP-1c expression, creates new perspectives aiming to unravel pathways by which PPAR*alpha* can protect the liver against lipogenesis induced by PPAR*gamma* overexpression as well as the precise mechanisms that underlie SREBP-1c and PPAR*gamma* interaction in the development of NAFLD.

The present study has certain limitations. The major aim of this study was to verify the efficiency of BZ to reverse the alterations caused by maternal diet-induced obesity in offspring's liver and WAT. Therefore, we examined the activation of PPAR*alpha* and PPAR*gamma* through BZ as well as proteins related to *beta*-oxidation and lipogenesis and metabolic parameters. Further studies are necessary to clarify separate actions of PPAR*alpha* and PPAR*gamma* in both organs as a *pair-feeding* group would enrich the discussion about BM loss and the role of PPARs in this context.

In conclusion, diet-induced maternal obesity lead to alterations in metabolism, hepatic lipotoxicity and adverse liver and adipose tissue remodeling caused in the offspring. Targeting PPAR by BZ has beneficial effects reducing the alterations, mainly through reduction of WAT inflammatory state through PPAR*gamma* activation and enhanced hepatic beta-oxidation due to increased PPAR*alpha*/PPAR*gamma* ratio in liver, resulting in higher expression of target genes involved with this pathway. The relevance of these observations stem from the fact that the effects of maternal obesity are not restricted to the first generation and might compromise future generations, leading to a continuation in increasing rates of obesity and DM2 worldwide.

## References

[1] Howie GJ, Sloboda DM, Kamal T, Vickers MH (2009) Maternal nutritional history predicts obesity in adult offspring independent of postnatal diet. J Physiol 587: 905–915. 19103681 10.1113/jphysiol.2008.163477 PMC2669979

[2] Drake AJ, Reynolds RM (2010) Impact of maternal obesity on offspring obesity and cardiometabolic disease risk. Reproduction 140: 387–398. 20562299 10.1530/REP-10-0077

[3] Gregorio BM, Souza-Mello V, Carvalho JJ, Mandarim-de-Lacerda CA, Aguila MB (2010) Maternal high-fat intake predisposes nonalcoholic fatty liver disease in C57BL/6 offspring. Am J Obstet Gynecol 203: 495 e491–498. 10.1016/j.ajog.2010.06.042 20822767

[4] Gustafson B, Hammarstedt A, Andersson CX, Smith U (2007) Inflamed adipose tissue: a culprit underlying the metabolic syndrome and atherosclerosis. Arterioscler Thromb Vasc Biol 27: 2276–2283. 17823366 10.1161/ATVBAHA.107.147835

[5] Catta-Preta M, Martins MA, Cunha Brunini TM, Mendes-Ribeiro AC, Mandarim-de-Lacerda CA, et al (2012) Modulation of cytokines, resistin, and distribution of adipose tissue in C57BL/6 mice by different high-fat diets. Nutrition 28: 212–219. 21872438 10.1016/j.nut.2011.05.011

[6] Fox CS, Massaro JM, Hoffmann U, Pou KM, Maurovich-Horvat P, et al (2007) Abdominal visceral and subcutaneous adipose tissue compartments: association with metabolic risk factors in the Framingham Heart Study. Circulation 116: 39–48. 17576866 10.1161/CIRCULATIONAHA.106.675355

[7] Pou KM, Massaro JM, Hoffmann U, Vasan RS, Maurovich-Horvat P, et al (2007) Visceral and subcutaneous adipose tissue volumes are cross-sectionally related to markers of inflammation and oxidative stress: the Framingham Heart Study. Circulation 116: 1234–1241. 17709633 10.1161/CIRCULATIONAHA.107.710509

[8] Eckel RH, Grundy SM, Zimmet PZ (2005) The metabolic syndrome. Lancet 365: 1415–1428. 15836891 10.1016/S0140-6736(05)66378-7

[9] Aguila M, Fernandes-Santos C, Pinheiro-Mulder A, Faria T, Mandarim-de-Lacerda C (2010) Hepatc insulin resistance and nonalcoholic fatty liver disease. In: J M, L G, editors. Insulin resistance : symptons, causes and treatment. New York: Nova Science pu. pp. 1–44.

[10] Brunt EM, Janney CG, Di Bisceglie AM, Neuschwander-Tetri BA, Bacon BR (1999) Nonalcoholic steatohepatitis: a proposal for grading and staging the histological lesions. Am J Gastroenterol 94: 2467–2474. 10484010 10.1111/j.1572-0241.1999.01377.x

[11] Festi D, Colecchia A, Sacco T, Bondi M, Roda E, et al (2004) Hepatic steatosis in obese patients: clinical aspects and prognostic significance. Obes Rev 5: 27–42. 14969505 10.1111/j.1467-789x.2004.00126.x

[12] Souza-Mello V, Mandarim-de-Lacerda CA, Aguila MB (2007) Hepatic structural alteration in adult programmed offspring (severe maternal protein restriction) is aggravated by post-weaning high-fat diet. Br J Nutr 98: 1159–1169. 17559700 10.1017/S0007114507771878

[13] Christodoulides C, Vidal-Puig A (2010) PPARs and adipocyte function. Mol Cell Endocrinol 318: 61–68. 19772894 10.1016/j.mce.2009.09.014

[14] Takahashi S, Tanaka T, Kodama T, Sakai J (2006) Peroxisome proliferator-activated receptor delta (PPARdelta), a novel target site for drug discovery in metabolic syndrome. Pharmacol Res 53: 501–507. 16713711 10.1016/j.phrs.2006.03.019

[15] Peters JM, Aoyama T, Burns AM, Gonzalez FJ (2003) Bezafibrate is a dual ligand for PPARalpha and PPARbeta: studies using null mice. Biochim Biophys Acta 1632: 80–89. 12782154 10.1016/s1388-1981(03)00065-9

[16] Tenenbaum H, Behar S, Boyko V, Adler Y, Fisman EZ, et al (2007) Long-term effect of bezafibrate on pancreatic beta-cell function and insulin resistance in patients with diabetes. Atherosclerosis 194: 265–271. 16970952 10.1016/j.atherosclerosis.2006.08.005

[17] Tenenbaum A, Fisman EZ, Motro M, Adler Y (2008) Optimal management of combined dyslipidemia: what have we behind statins monotherapy? Adv Cardiol 45: 127–153. 18230960 10.1159/000115192

[18] Nagasawa T, Inada Y, Nakano S, Tamura T, Takahashi T, et al (2006) Effects of bezafibrate, PPAR pan-agonist, and GW501516, PPARdelta agonist, on development of steatohepatitis in mice fed a methionine- and choline-deficient diet. Eur J Pharmacol 536: 182–191. 16574099 10.1016/j.ejphar.2006.02.028

[19] Reagan-Shaw S, Nihal M, Ahmad N (2008) Dose translation from animal to human studies revisited. FASEB J 22: 659–661. 17942826 10.1096/fj.07-9574LSF

[20] Barbosa-da-Silva S, Fraulob-Aquino JC, Lopes JR, Mandarim-de-Lacerda CA, Aguila MB (2012) Weight cycling enhances adipose tissue inflammatory responses in male mice. PLoS One 7: e39837. 22848362 10.1371/journal.pone.0039837 PMC3405086

[21] Matthews DR, Hosker JP, Rudenski AS, Naylor BA, Treacher DF, et al (1985) Homeostasis model assessment: insulin resistance and beta-cell function from fasting plasma glucose and insulin concentrations in man. Diabetologia 28: 412–419. 3899825 10.1007/BF00280883

[22] Tschanz SA, Burri PH, Weibel ER (2011) A simple tool for stereological assessment of digital images: the STEPanizer. J Microsc 243: 47–59. 21375529 10.1111/j.1365-2818.2010.03481.x

[23] Catta-Preta M, Mendonca LS, Fraulob-Aquino J, Aguila MB, Mandarim-de-Lacerda CA (2011) A critical analysis of three quantitative methods of assessment of hepatic steatosis in liver biopsies. Virchows Arch 459: 477–485. 21901430 10.1007/s00428-011-1147-1

[24] Souza-Mello V, Gregorio BM, Cardoso-de-Lemos FS, de Carvalho L, Aguila MB, et al (2010) Comparative effects of telmisartan, sitagliptin and metformin alone or in combination on obesity, insulin resistance, and liver and pancreas remodelling in C57BL/6 mice fed on a very high-fat diet. Clin Sci (Lond) 119: 239–250. 20415664 10.1042/CS20100061

[25] Samuelsson AM, Matthews PA, Argenton M, Christie MR, McConnell JM, et al (2008) Diet-induced obesity in female mice leads to offspring hyperphagia, adiposity, hypertension, and insulin resistance: a novel murine model of developmental programming. Hypertension 51: 383–392. 18086952 10.1161/HYPERTENSIONAHA.107.101477

[26] Sullivan EL, Smith MS, Grove KL (2011) Perinatal exposure to high-fat diet programs energy balance, metabolism and behavior in adulthood. Neuroendocrinology 93: 1–8. 21079387 10.1159/000322038 PMC3700139

[27] Fernandes-Santos C, Carneiro RE, de Souza Mendonca L, Aguila MB, Mandarim-de-Lacerda CA (2009) Pan-PPAR agonist beneficial effects in overweight mice fed a high-fat high-sucrose diet. Nutrition 25: 818–827. 19268533 10.1016/j.nut.2008.12.010

[28] Vickers MH, Reddy S, Ikenasio BA, Breier BH (2001) Dysregulation of the adipoinsular axis – a mechanism for the pathogenesis of hyperleptinemia and adipogenic diabetes induced by fetal programming. J Endocrinol 170: 323–332. 11479129 10.1677/joe.0.1700323

[29] De Souza CT, Frederico MJ, da Luz G, Cintra DE, Ropelle ER, et al (2010) Acute exercise reduces hepatic glucose production through inhibition of the Foxo1/HNF-4alpha pathway in insulin resistant mice. J Physiol 588: 2239–2253. 20421289 10.1113/jphysiol.2009.183996 PMC2911223

[30] Teramoto T, Shirai K, Daida H, Yamada N (2012) Effects of bezafibrate on lipid and glucose metabolism in dyslipidemic patients with diabetes: the J-BENEFIT study. Cardiovasc Diabetol 11: 29. 22439599 10.1186/1475-2840-11-29 PMC3342914

[31] Kwon EY, Shin SK, Cho YY, Jung UJ, Kim E, et al (2012) Time-course microarrays reveal early activation of the immune transcriptome and adipokine dysregulation leads to fibrosis in visceral adipose depots during diet-induced obesity. BMC Genomics 13: 450. 22947075 10.1186/1471-2164-13-450 PMC3447724

[32] Lee JK, Seo EM, Lee SS, Park SH, Sim YB, et al (2010) Activation of PPARalpha Attenuates IFNgamma and IL-1beta-induced Cell Proliferation in Astrocytes: Involvement of IL-6 Independent Pathway. Korean J Physiol Pharmacol 14: 185–189. 20631892 10.4196/kjpp.2010.14.3.185 PMC2902811

[33] Kleemann R, Gervois PP, Verschuren L, Staels B, Princen HM, et al (2003) Fibrates down-regulate IL-1-stimulated C-reactive protein gene expression in hepatocytes by reducing nuclear p50-NFkappa B-C/EBP-beta complex formation. Blood 101: 545–551. 12393563 10.1182/blood-2002-06-1762

[34] Steppan CM, Bailey ST, Bhat S, Brown EJ, Banerjee RR, et al (2001) The hormone resistin links obesity to diabetes. Nature 409: 307–312. 11201732 10.1038/35053000

[35] Okuno A, Tamemoto H, Tobe K, Ueki K, Mori Y, et al (1998) Troglitazone increases the number of small adipocytes without the change of white adipose tissue mass in obese Zucker rats. J Clin Invest 101: 1354–1361. 9502777 10.1172/JCI1235 PMC508690

[36] Goto T, Lee JY, Teraminami A, Kim YI, Hirai S, et al (2011) Activation of peroxisome proliferator-activated receptor-alpha stimulates both differentiation and fatty acid oxidation in adipocytes. J Lipid Res 52: 873–884. 21324916 10.1194/jlr.M011320 PMC3073464

[37] Berg AH, Combs TP, Scherer PE (2002) ACRP30/adiponectin: an adipokine regulating glucose and lipid metabolism. Trends Endocrinol Metab 13: 84–89. 11854024 10.1016/s1043-2760(01)00524-0

[38] Kudoh A, Satoh H, Hirai H, Watanabe T (2011) Pioglitazone upregulates adiponectin receptor 2 in 3T3-L1 adipocytes. Life sci 88 23–24: 1055–62. 21514306 10.1016/j.lfs.2011.04.001

[39] Hays T, Rusyn I, Burns AM, Kennett MJ, Ward JM, et al (2005) Role of peroxisome proliferator-activated receptor-alpha (PPARalpha) in bezafibrate-induced hepatocarcinogenesis and cholestasis. Carcinogenesis 26: 219–227. 15447978 10.1093/carcin/bgh285

[40] Berger Ma (2002) The mechanisms of action of PPARs. Annu Rev Med 53: 409–435. 11818483 10.1146/annurev.med.53.082901.104018

[41] Rakhshandehroo M, Hooiveld G, Muller M, Kersten S (2009) Comparative analysis of gene regulation by the transcription factor PPARalpha between mouse and human. PLoS One 4: e6796. 19710929 10.1371/journal.pone.0006796 PMC2729378

[42] Desvergne B, Wahli W (1999) Peroxisome proliferator-activated receptors: nuclear control of metabolism. Endocr Rev 20: 649–688. 10529898 10.1210/edrv.20.5.0380

[43] Tenenbaum A, Motro M, Fisman EZ (2005) Dual and pan-peroxisome proliferator-activated receptors (PPAR) co-agonism: the bezafibrate lessons. Cardiovasc Diabetol 4: 14. 16168052 10.1186/1475-2840-4-14 PMC1236941

[44] Lee HJ, Choi SS, Park MK, An YJ, Seo SY, et al (2002) Fenofibrate lowers abdominal and skeletal adiposity and improves insulin sensitivity in OLETF rats. Biochem Biophys Res Commun 296: 293–299. 12163016 10.1016/s0006-291x(02)00822-7

[45] Serviddio G, Giudetti AM, Bellanti F, Priore P, Rollo T, et al (2011) Oxidation of hepatic carnitine palmitoyl transferase-I (CPT-I) impairs fatty acid beta-oxidation in rats fed a methionine-choline deficient diet. PLoS One 6: e24084. 21909411 10.1371/journal.pone.0024084 PMC3164715

[46] Pettinelli P, Videla LA (2011) Up-regulation of PPAR-gamma mRNA expression in the liver of obese patients: an additional reinforcing lipogenic mechanism to SREBP-1c induction. J Clin Endocrinol Metab 96: 1424–1430. 21325464 10.1210/jc.2010-2129

[47] Chen G, Liang G, Ou J, Goldstein JL, Brown MS (2004) Central role for liver X receptor in insulin-mediated activation of Srebp-1c transcription and stimulation of fatty acid synthesis in liver. Proc Natl Acad Sci U S A 101: 11245–11250. 15266058 10.1073/pnas.0404297101 PMC509189

[48] Campbell SE, Tandon NN, Woldegiorgis G, Luiken JJ, Glatz JF, et al (2004) A novel function for fatty acid translocase (FAT)/CD36: involvement in long chain fatty acid transfer into the mitochondria. J Biol Chem 279: 36235–36241. 15161924 10.1074/jbc.M400566200

[49] Vosper H, Patel L, Graham TL, Khoudoli GA, Hill A, et al (2001) The peroxisome proliferator-activated receptor delta promotes lipid accumulation in human macrophages. J Biol Chem 276: 44258–44265. 11557774 10.1074/jbc.M108482200

[50] Cabrero A, Cubero M, Llaverias G, Jove M, Planavila A, et al (2003) Differential effects of peroxisome proliferator-activated receptor activators on the mRNA levels of genes involved in lipid metabolism in primary human monocyte-derived macrophages. Metabolism 52: 652–657. 12759900 10.1053/meta.2003.50100

[51] Pettinelli P, Del Pozo T, Araya J, Rodrigo R, Araya AV, et al (2009) Enhancement in liver SREBP-1c/PPAR-alpha ratio and steatosis in obese patients: correlations with insulin resistance and n-3 long-chain polyunsaturated fatty acid depletion. Biochim Biophys Acta 1792: 1080–1086. 19733654 10.1016/j.bbadis.2009.08.015

[52] Brown MS, Goldstein JL (1997) The SREBP pathway: regulation of cholesterol metabolism by proteolysis of a membrane-bound transcription factor. Cell 89: 331–340. 9150132 10.1016/s0092-8674(00)80213-5

[53] Liang G, Yang J, Horton JD, Hammer RE, Goldstein JL, et al (2002) Diminished hepatic response to fasting/refeeding and liver X receptor agonists in mice with selective deficiency of sterol regulatory element-binding protein-1c. J Biol Chem 277: 9520–9528. 11782483 10.1074/jbc.M111421200

[54] Gavrilova O, Haluzik M, Matsusue K, Cutson JJ, Johnson L, et al (2003) Liver peroxisome proliferator-activated receptor gamma contributes to hepatic steatosis, triglyceride clearance, and regulation of body fat mass. J Biol Chem 278: 34268–34276. 12805374 10.1074/jbc.M300043200

[55] Way JM, Harrington WW, Brown KK, Gottschalk WK, Sundseth SS, et al (2001) Comprehensive messenger ribonucleic acid profiling reveals that peroxisome proliferator-activated receptor gamma activation has coordinate effects on gene expression in multiple insulin-sensitive tissues. Endocrinology 142: 1269–1277. 11181544 10.1210/endo.142.3.8037

